# Weakly basic electrolysed water combined with ultrasound for enhancing the binding and functional properties of Antarctic krill protein–gallic acid complexes

**DOI:** 10.1016/j.ultsonch.2026.108036

**Published:** 2026-09-04

**Authors:** Jinsong Wang, Deyan Zhu, Rong Li, Yijiao Sun, Yufeng Li

**Affiliations:** a Team for Food Nutritional Regulation and Innovation, Hubei Academy of New Rural Development, Jingchu University of Technology, Jingmen 448000, China; b Hubei Key Laboratory of Drug Synthesis and Optimization, Jingchu University of Technology, Jingmen 448000, China; c College of Biological and Food Engineering, Anhui Polytechnic University, Wuhu 241000, China; d College of Food and Biology, Jingchu University of Technology, Jingmen 448000, China

**Keywords:** Antarctic krill protein, Gallic acid, Weakly basic electrolysed water, Emulsification, Antioxidant

## Abstract

Ultrasonic treatment is an effective strategy for modifying protein structure. However, the accompanying generation of reactive oxygen species (ROS) inevitably induces protein oxidation and polyphenol degradation, compromising the functional performance of protein–polyphenol complexes. This study combined weakly basic electrolysed water (WBEW) with ultrasonic treatment to investigate its protective effects on the complexation between Antarctic krill protein and gallic acid (GA), as well as the structural and functional properties of the resulting complexes. WBEW effectively quenched ultrasound-generated •OH, reducing •OH levels from 4.36 to 3.02 μmol/L after 40 min of ultrasonication. Consequently, WBEW significantly suppressed protein carbonylation and sulphydryl loss and notably enhanced GA retention. A maximum binding efficiency of 34.8% was achieved at 20 min of ultrasonication in WBEW, corresponding to optimal particle size reduction (202.3 nm), improved solubility, and superior surface hydrophobicity. Fourier-transform infrared and fluorescence spectroscopy revealed that WBEW alleviated ultrasound-induced secondary and tertiary structural perturbations, maintaining more ordered conformations. The WBEW-20 complex exhibited significantly increased emulsifying activity (40.3  m^2^/g) and emulsifying stability indices, enabling the formation of high-internal-phase emulsions with uniform droplet size and thick interfacial layers. Moreover, emulsions stabilised by the WBEW-20 complex demonstrated remarkably lower peroxide (20.1 mmol/kg) and malondialdehyde (4.34 mmol/kg) values after 11 days of storage than the controls, along with superior thermal and ultraviolet oxidation resistance. These findings establish WBEW-assisted ultrasound treatment as a promising modification strategy for upgrading marine protein–polyphenol complexes with enhanced emulsifying and antioxidant functionalities for application in oxidation-sensitive food systems.

## 1 Introduction

Antarctic krill (*Euphausia superba*) represents a significant, yet largely underutilised, marine bioresource. Despite annual global catches exceeding 300,000 t, most krill is currently processed into low-value animal feed rather than products for human consumption [1]. While Antarctic krill proteins (AKPs) offer a balanced amino acid profile and high digestibility, their use in food is limited by poor solubility, especially near their isoelectric point (pH 4–5). Furthermore, their compact globular structure and susceptibility to aggregation during processing result in inadequate emulsifying capacity, foaming stability, and thermal gelation properties [2]. Complexing proteins with dietary polyphenols has emerged as an effective, environmentally friendly approach to enhance both the techno-functional and nutritional attributes of proteins [3]. Gallic acid (GA)—a simple yet potent natural phenolic acid found in fruits, tea, and wine—is particularly promising. It possesses strong electron-donating capabilities and well-documented bioactivities, including anti-inflammatory, antimicrobial, and cardioprotective effects. The interaction between GA and proteins, whether covalent or non-covalent, can significantly alter protein secondary and tertiary structures. This exposure of hydrophobic cores and introduction of phenolic hydroxyl groups into the protein matrix can lead to improved solubility, interfacial properties, thermal stability, and overall antioxidant activity of the resulting complexes [4]. Concurrently, protein complexation acts as an effective carrier system, protecting GA from environmental degradation and potentially enhancing its bioaccessibility in the gastrointestinal tract, thereby creating a mutually beneficial synergistic system.

Ultrasound treatment is increasingly recognised as an efficient physical method for facilitating protein–polyphenol association [5]. Acoustic cavitation generates intense shear forces, microstreaming, and turbulent flow, which mechanically disrupt aggregated protein structures. This promotes the exposure of previously buried hydrophobic patches and reactive nucleophilic residues (e.g., thiol and amino groups), which serve as primary binding sites for polyphenols. Nevertheless, these same cavitation events also induce sonochemical effects. The pyrolytic dissociation of water molecules within collapsing bubbles produces highly reactive oxygen species (ROS), predominantly hydroxyl radicals (•OH) and singlet oxygen (^1^O_2_) [6]. These radicals can aggressively oxidise sulphur-containing and aromatic amino acid residues (methionine, cysteine, tryptophan, and tyrosine), leading to carbonyl derivative formation, sulphydryl group depletion, and protein cross-linking or fragmentation [5]. Simultaneously, the phenolic hydroxyl groups of GA are vulnerable to radical attack, resulting in oxidative degradation and polymerisation, which reduces the number of intact polyphenol molecules available for binding [7].

Weakly basic electrolysed water (WBEW), typically produced by electrolysing a dilute salt solution in a non-membrane electrolytic cell, is characterised by a mild pH range of 9.0–10.0 and a highly negative oxidation–reduction potential (ORP). This endows it with remarkable reducing capacity and potent free-radical scavenging activity [8]. When applied during ultrasound processing, WBEW’s strong electron-donating ability can effectively quench the •OH and 1O_2_ generated in situ, thereby protecting the reactive groups on both protein side chains and GA from oxidative modification [9]. Concurrently, its mild alkaline environment may subtly modulate the electrostatic charge distribution on the protein surface, potentially enhancing electrostatic attraction towards the negatively charged GA, while its buffering capacity prevents drastic pH fluctuations that could otherwise cause protein precipitation [10]. These findings suggest that WBEW may serve as an ideal medium to reconcile the contradictory effects of ultrasound, harnessing its structural modification benefits while suppressing its oxidative drawbacks.

Our previous work demonstrated that basic electrolysed water combined with ultrasound could modulate the conformation, solubility and digestibility of Antarctic krill protein [11]. Our previous work demonstrated that basic electrolysed water combined with ultrasound could modulate the conformation, solubility and digestibility of Antarctic krill protein. Nevertheless, it remains unknown whether WBEW can regulate protein–polyphenol binding behaviour under ultrasonic cavitation conditions. During ultrasound–assisted complex preparation, sonochemically generated ROS can cause dual oxidative damage: it triggers oxidation of amino–acid residues on AKP, and simultaneously induces the degradation of gallic acid. Such oxidative side–effects would decrease effective binding sites and reduce the amount of intact gallic–acid available for interaction, ultimately lowering binding efficiency and deteriorating the functionalities of AKP–GA complexes.

We hypothesised that incorporating WBEW into the ultrasound processing system would first effectively scavenge sonochemically–derived •OH and other reactive oxygen species. This ROS suppression protects oxidation–prone reactive thiol and amino groups on AKP, as well as phenolic hydroxyl moieties on GA from radical–mediated degradation. Preserving these reactive chemical groups maintains abundant available sites for covalent and non–covalent interactions between AKP and GA. Meanwhile, the mild–alkaline environment of WBEW modulates surface electrostatic charge of AKP to favour attraction towards negatively–charged GA. Collectively, these combined effects improve the binding efficiency of AKP–GA complexes while retaining the beneficial conformational unfolding effects induced by ultrasonic cavitation. Accordingly, the present study systematically evaluated the effects of WBEW on the binding efficiency, structural characteristics, surface hydrophobicity, and functional performance (solubility, emulsifying activity, and antioxidant capacity) of the resulting AKP–GA complexes, as well as the lipid–oxidation resistance of high–internal–phase emulsions stabilised by these complexes.

## 2 Materials and methods

### 2.1 Materials

GA (purity ≥ 98%), 5,5-dithio-bis-(2-nitrobenzoic acid) (DTNB), 2,4-dinitrophenylhydrazine (DNPH), trichloroacetic acid (TCA), and thiobarbituric acid were procured from Sigma-Aldrich Co., Ltd. (Shanghai, China). Food-grade soybean oil was purchased from a local supermarket. Sodium dihydrogen phosphate and disodium hydrogen phosphate (analytical grade) were used for phosphate buffer solution (PBS) preparation. All other chemical reagents were of analytical grade.

### 2.2 Extraction of Antarctic krill proteins

The Antarctic krill protein was extracted according to our previous study with slight modification [11]. Antarctic krill meat was mixed with deionized water in a proportion of 1:10. The abovementioned mixture was then homogenized at 300 rpm. After that, the pH value of the homogenate was adjusted to 12 by NaOH (2 mol/L). The mixture was stirred for 2 h, followed by centrifugation at 10000 × g for 8 min at 4 ℃. The pH value of the supernatant was adjusted to 4.5 with HCl (2 mol/L). Subsequently, the solution was centrifuged to obtain crude AKPs. The harvested AKPs were further purified by a three-stage countercurrent washing method and freeze-dried to obtain pure AKP powder for subsequent experiments. Finally, the protein content (80.12%) of the prepared sample was quantitatively determined by combustion method using a Kjeldahl nitrogen determinator. Prior to use, the AKP powder was further defatted twice with n–hexane to eliminate interferences induced by residual lipids in subsequent spectral measurements.

### 2.3 Preparation of WBEW

WBEW was generated using a laboratory electrolysed water machine (FW-200 (AMANO Cor poration, Kanagawa, Japan), following the method described by Li et al. [11] with slight modifications. Briefly, a low-concentration NaCl solution (0.05%, w/v) was prepared in deionised water and used as the electrolyte. The electrolysis was performed under constant current mode with the following parameters: applied voltage, 220.0 ± 0.5 V; electrolysis duration = 5 min. The obtained WBEW exhibited a stable alkaline pH (9.0 ± 0.2) and negative ORP (−220 to − 230 mV).

### 2.4 Ultrasound-assisted preparation of AKP–GA complexes

AKP powder was dissolved in either deionised water (DW) or WBEW to a final concentration of 10 mg/mL. This solution was stirred overnight at 4 °C to ensure complete hydration. GA was subsequently added to the protein solution at a mass ratio of 1:20 (GA: AKP). The mixture underwent ultrasonic treatment using a probe-type sonicator (Scientz-IID; Ningbo Scientz Biotechnology Co., Ltd., China) fitted with a 6-mm titanium probe. The ultrasonic parameters were set as follows: power, 300 W; frequency, 20 kHz; and pulse duration, 5 s on and 5 s off. Treatment durations were 0, 10, 20, 30, and 40 min. To maintain the temperature below 20 °C during sonication, the sample beaker was immersed in an ice-water bath. Following treatment, the solutions were centrifuged, freeze-dried, and stored at − 20 °C for subsequent structural and functional analysis. The resulting complexes were designated as DW-0 to DW-40 or WBEW-0 to WBEW-40, where the number indicates the ultrasonic treatment time (0, 10, 20, 30, or 40 min) in DW or WBEW, respectively.

### 2.5 Determination of •OH and ORP

The concentration of •OH generated during ultrasound treatment was determined using the terephthalic acid (TA) fluorescence probe method. Prior to sonication, 0.5 mM TA was added to the sample solutions. After ultrasonic treatment, the fluorescence intensity of the resulting 2-hydroxyterephthalic acid was measured at excitation and emission wavelengths of 315 and 425 nm, respectively, using a fluorescence spectrophotometer (F-7000; Hitachi, Japan). The •OH concentration was calculated against a standard curve was built with serial concentrations of authentic 2–hydroxyterephthalic acid standard. The ORP of the protein solutions was measured before and after ultrasound treatment using a calibrated ORP electrode (Orion Star A221; Thermo Fisher Scientific, USA) at 25 °C [8].

### 2.6 Determination of protein carbonyl and free sulfhydryl contents

Protein carbonyl content was determined using the DNPH method ^8^. Briefly, protein samples (2 mg/mL) were reacted with 10 mM DNPH in 2 M HCl for 1 h at 25 °C in the dark. After precipitation with 20% TCA, the protein pellets were washed three times with ethanol/ethyl acetate (1:1, v/v), then dissolved in 6 M guanidine hydrochloride. Absorbance was measured at 370 nm, and carbonyl content was calculated using a molar extinction coefficient of 22,000 M^−1^ cm^−1^.

Free sulphydryl content was determined via the DTNB colorimetric method. Protein samples were mixed with Tris-Gly buffer and DTNB working solution, and incubated in the dark, with absorbance measured at 412 nm. The free sulphydryl content was calculated from a standard curve and expressed as µmol/g protein.

### 2.7 GA retention assay

The GA retention rate in the supernatant post-ultrasound treatment was determined using the Folin–Ciocalteu method. Briefly, 0.2 mL of the sample was combined with 1 mL of Folin–Ciocalteu reagent (1:10 dilution) and 0.8 mL of 7.5% sodium carbonate solution. After a 30-min incubation in the dark at 25 °C, absorbance was measured at 760 nm. The total phenolic content was calculated against a GA standard curve.

### 2.8 Conjugation degree between AKP and GA

The degree of conjugation between AKP and GA was determined by measuring the amount of GA bound to proteins after dialysis. Free GA was separated from protein-bound GA by dialysing against the appropriate solvent (DW or WBEW) at 4 °C for 48 h, with multiple changes of the dialysis buffer. The total phenolic content in the dialysed protein solution was subsequently measured using the Folin–Ciocalteu method, as previously described. Binding efficiency was calculated using Eq. (1):

(1) $$\;\;\;\;\;\;\;\;\;Conjugationdegree=\frac{\mathrm{boundGA}}{\mathrm{initialgallicGA}}\times \;100$$

### 2.9 Determination of particle size and solubility of modified AKP

The hydrodynamic particle size of AKP in different treatment groups was determined using a dynamic light scattering (DLS) particle size analyser (Zetasizer Nano ZS; Malvern Instruments, UK). The solubility of AKP (1 mg/mL) was determined via centrifugation. The protein dispersion was centrifuged at 8, 000 r/min for 15 min. The protein content of the supernatant and the total protein content of the original solution were subsequently determined using the Coomassie brilliant blue method. GA–only blank controls with identical GA concentration and corresponding ultrasonic treatment were prepared in parallel. Absorbance values of GA blanks were subtracted during protein quantification to eliminate spectral interferences derived from free GA for Coomassie brilliant blue assay. Protein solubility was calculated as the ratio of supernatant protein content to total protein content [2].

### 2.10 Scanning electron microscopy (SEM) analysis

The micromorphology of freeze-dried AKP samples was observed using a scanning electron microscope (SU8010; Hitachi, Japan). The dried powder was uniformly adhered to conductive adhesive, gold-sprayed, and observed under an accelerating voltage of 5 kV.

### 2.11 Atomic force microscopy (AFM) analysis

The morphological features of AKP samples in their hydrated state were visualised using atomic force microscopy (Bruker Dimension Icon, Germany). Protein samples were diluted to 10 μg/mL with 10 mM phosphate buffer (pH 7.0). A 10 μL aliquot of the diluted sample was deposited onto a freshly cleaved mica substrate and allowed to adsorb for 5 min, followed by gentle rinsing with ultrapure water and drying under a nitrogen stream. AFM imaging was performed in tapping mode using silicon cantilevers.

### 2.12 FT-IR spectral analysis and protein secondary structure quantification

The FT-IR spectra of AKP–GA complexes were recorded using an FT-IR spectrometer [1]. The freeze-dried sample powder was mixed with KBr at a mass ratio of 1:100 and pressed into transparent flakes. The scanning range was 4,000–400 cm^−1^ with a resolution of 4 cm^−1^ and 32 scans. The amide I band (1,700–1,600 cm^−1^) was analysed for secondary structure estimation using PeakFit v4.12 software. The relative contents of α-helix, β-sheet, β-turn, and random coil were quantitatively calculated based on the peak area ratio of each secondary structure component.

### 2.13 Endogenous fluorescence spectroscopy measurement

The endogenous fluorescence spectrum of AKP–GA complexes was determined using a fluorescence spectrophotometer (F-7000; Hitachi, Japan). The excitation wavelength was set at 280 nm, and the emission scanning range was 290–450 nm. The slit width and scanning speed were 5 nm and 1,200 nm/min, respectively. Parallel GA–only blank samples with the same GA concentration and corresponding ultrasonic treatment were measured under identical instrument settings. Fluorescence signals from GA blanks were subtracted from the raw spectra of AKP–GA complexes to eliminate background noise and inner–filter effects induced by gallic acid under 280  nm excitation. The maximum emission wavelength and fluorescence intensity were recorded [11].

### 2.14 Surface hydrophobicity measurement

Surface hydrophobicity (*H_0_*) was determined using the 8-anilino-1-naphthalenesulfonic acid (ANS) fluorescence probe method [12]. Protein samples were serially diluted to concentrations of 0.05–0.5 mg/mL in 10 mM phosphate buffer (pH 7.0). An 8 mM ANS solution, prepared in phosphate buffer, was added to each dilution at a 1:50 vol ratio (ANS:protein). After a 15-min incubation at 25 °C in the dark, fluorescence intensity was measured with excitation and emission wavelengths set at 390 and 470 nm, respectively. Corresponding GA–only blank controls were prepared and measured under identical experimental conditions. *H_0_* was calculated as the initial slope of fluorescence intensity versus protein concentration.

### 2.15 Contact angle measurement

The contact angles of protein films were measured using a DSA30 contact angle goniometer (Krüss, Germany). Freeze-dried protein samples were compressed into flat tablets (10 mm diameter, 2 mm thickness) using a hydraulic press. A 5 μL droplet of DW was placed on the tablet surface, and the contact angle was measured immediately. Five measurements were performed for each sample [13].

### 2.16 Emulsifying activity index (EAI) and emulsion stability index (ESI)

The emulsifying properties of AKP–gallic acid complexes were evaluated according to the method of Shi et al. [14]. Protein solutions (2 wt%, in phosphate buffer, 10 mM, pH 7.0) were mixed with soybean oil at a volume ratio of 1:3(protein:oil) and homogenized using a high-speed homogenizer (T25, IKA, Germany) at 10,000 rpm for 1 min. An aliquot (50 μL) of the emulsion was immediately taken from the bottom of the beaker and diluted with 5 mL of 0.1% SDS solution. The absorbance at 500 nm was measured at 0 min (A_0_) and after 10 min (A_1_*_0_*). EAI and ESI were calculated as follows:

(2) $$\;\;\;\;\;\;\;\;\;\;\;\;\;\;\;\;\;\;\;\;EAI\left(\frac{m^{2}}{g}\right)=\frac{2\times 2.303}{C\times (1-\varphi )\times 10000}\times A_{0}\times D$$

(3) $$\;\;\;\;\;\;\;\;\;\;\;\;\;\;\;\;\;\;\;\;ESI\left(min\right)=\frac{A_{0}}{A_{0}-A_{10}}\times 10$$

where *C* represent the weight of protein per volume (g/mL); *φ* represents the volume fraction of oil in the emulsion(0.75); D represents the dilution factor, respectively.

### 2.17 Confocal laser scanning microscopy (CLSM) analysis

High-internal-phase emulsions (HIPEs) were fabricated using untreated AKP, DW-20, and WBEW-20 modified AKP–GA complexes as emulsifiers. Their microstructure was observed using an LSM 880 confocal laser scanning microscope (Zeiss, Germany). Oil droplets were stained with Nile Red (1 mg/mL in acetone), and proteins were stained with Nile Blue (1 mg/mL in water). The stained emulsions were placed on glass slides and observed at excitation wavelengths of 488 nm (Nile Red) and 633 nm (Nile Blue) [2].

### 2.18 Determination of lipid oxidation indices

The prepared high-internal-phase emulsions were placed under conventional storage, thermal treatment (70 ℃) and UV irradiation conditions for accelerated oxidation. During storage, emulsion samples were taken at regular intervals. The peroxide value (POV) was determined by the iodometric titration method, and the malondialdehyde (MDA) content was measured by the TBA colorimetric method. The primary and secondary lipid oxidation degrees of emulsions stabilized by different modified complexes were compared and analyzed.

### 2.19 Statistical analysis

All experiments were performed in triplicate, and results were expressed as mean ± standard deviation (SD). Statistical analysis was conducted using SPSS version 26.0 (IBM, USA). One-way analysis of variance (ANOVA) followed by Duncan’s multiple range test was used to determine significant differences between means at a confidence level of *p* < 0.05.

## 3 Results and discussion

### 3.1 WBEW alleviates ultrasound-induced oxidative damage and enhances binding efficiency

**Fig. 1:**
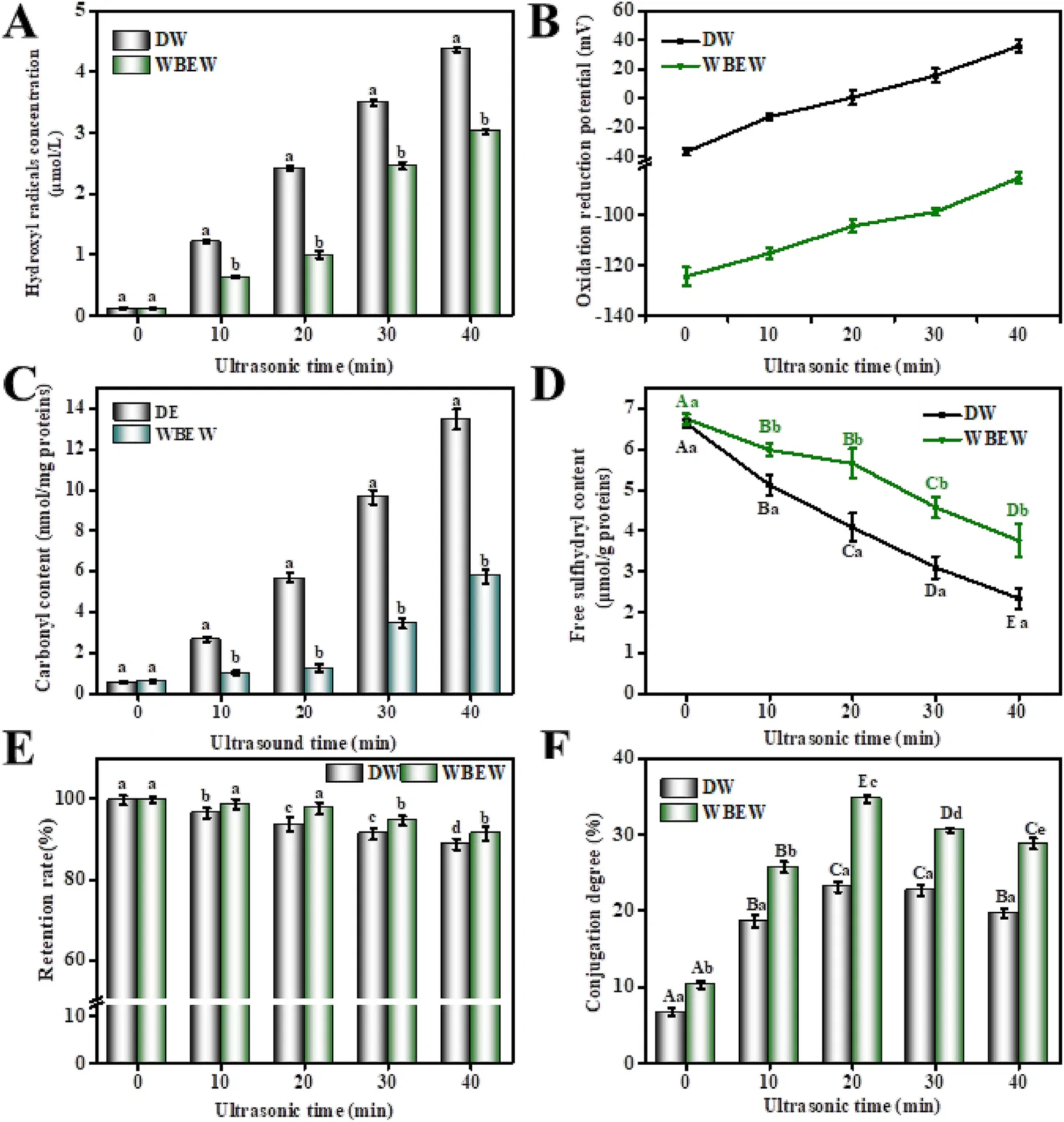
Changes in the yield of •OH radical (A), redox potential (B), carbonyl (C) and free sulfhydryl content (D) in AKP, retention rate of Gallic acid(E) and Conjugation degree of AKP and Gallic acid (F) after ultrasonic treatment for different time (0, 10, 20, 30 and 40 min). DW and WBEW represent treatment in DW and WBEW systems, respectively. Different letters (a, b, c, etc) indicate significant differences at *p* < 0.05.

To elucidate the protective role of WBEW in ultrasound-assisted protein–polyphenol complexation, we initially evaluated the oxidative environment and its effects on both protein integrity and polyphenol stability. As depicted in Fig. 1A, •OH generation increased with ultrasonic time in both DW and WBEW systems. Nonetheless, the WBEW system consistently produced significantly lower •OH levels, reaching only 3.02 μmol/L after 40 min compared to 4.36 μmol/L in DW. Correspondingly, the ORP of the AKP solution in WBEW remained markedly negative (−125.3 to − 82.4 mV) during ultrasound, while the DW system shifted from − 38.2 to − 37.6 mV (Fig. 1B). The strong reducing capacity of WBEW, attributed to its negative ORP and active hydrogen species, effectively quenches the reactive radicals generated by cavitation, thus providing a protective microenvironment [8].

Protein carbonyl and free sulfhydryl contents are established biomarkers for assessing protein oxidative modification [11]. In Fig. 1C, prolonged ultrasonic treatment markedly increased the carbonyl content of AKP in both systems, stemming from ROS-induced oxidation of amino acid side chains and peptide backbone cleavage during cavitation. Nevertheless, the WBEW group exhibited significantly lower carbonyl levels than the DW group at identical ultrasonic times, demonstrating WBEW’s effective suppression of ultrasonic-triggered protein carbonylation. For free sulphydryl groups (Fig. 1D), ultrasonic oxidation led to a continuous loss of sulphydryl residues in both groups. The free sulphydryl content of DW-treated AKP sharply decreased from 4.6 to 1.3 μmol/g protein after 40 min of ultrasonication, while that of WBEW-treated AKP only declined from 4.7 to 2.5 μmol/g protein. Ultrasonic cavitation generates various ROS, including ·OH, superoxide anion radicals, and singlet oxygen, which readily oxidise nucleophilic sulphydryl groups, inducing disulphide cross-linking and protein aggregation [15], [16]. The significantly higher sulphydryl retention in the WBEW group further substantiates WBEW’s ROS-scavenging and antioxidant functions, which protected the binding sites of AKP for polyphenol grafting.

The protective effect of WBEW on polyphenol stability was further validated by the GA retention rate (Fig. 1E). Ultrasonic ROS inevitably induced oxidative degradation of GA, leading to a gradual decrease in polyphenol retention with prolonged treatment time in both systems. Notably, the WBEW system maintained a distinctly higher GA retention rate than the DW group under all ultrasonic conditions. This is attributed to the susceptibility of GA’s phenolic hydroxyl groups to radical-mediated degradation; WBEW’s radical scavenging ability preserves intact polyphenol molecules, which are essential for effective protein binding [17].

The binding efficiency between AKP and GA further elucidated the synergistic modification of ultrasound combined with WBEW (Fig. 1F). Ultrasonic pretreatment consistently improved the binding degree of AKP and polyphenols in both systems. This enhancement emanates from ultrasonic mechanical cavitation, which unfolds the AKP conformation and exposes more hidden binding sites. The WBEW group achieved a maximum binding efficiency of 34.8% after 20 min of ultrasonication. However, excessive ultrasonic treatment (exceeding 20 min) elicited a gradual decline in conjugation efficiency. This decline is primarily due to the excessive accumulation of ROS during prolonged ultrasonication, which triggers dual oxidation of AKP active residues and GA active structures, thereby reducing the effective binding probability between the protein and polyphenol [18], [19]. Compared with the DW group, the WBEW group consistently exhibited significantly higher binding efficiency across all matched time points. These results firmly establish that WBEW substantially mitigates ultrasound-induced oxidation of AKPs and GA, consequently enhancing the net binding efficiency, with 20 min identified as the optimal treatment duration.

### 3.2 Effects of WBEW on protein solubility and particle sizes

**Fig. 2:**
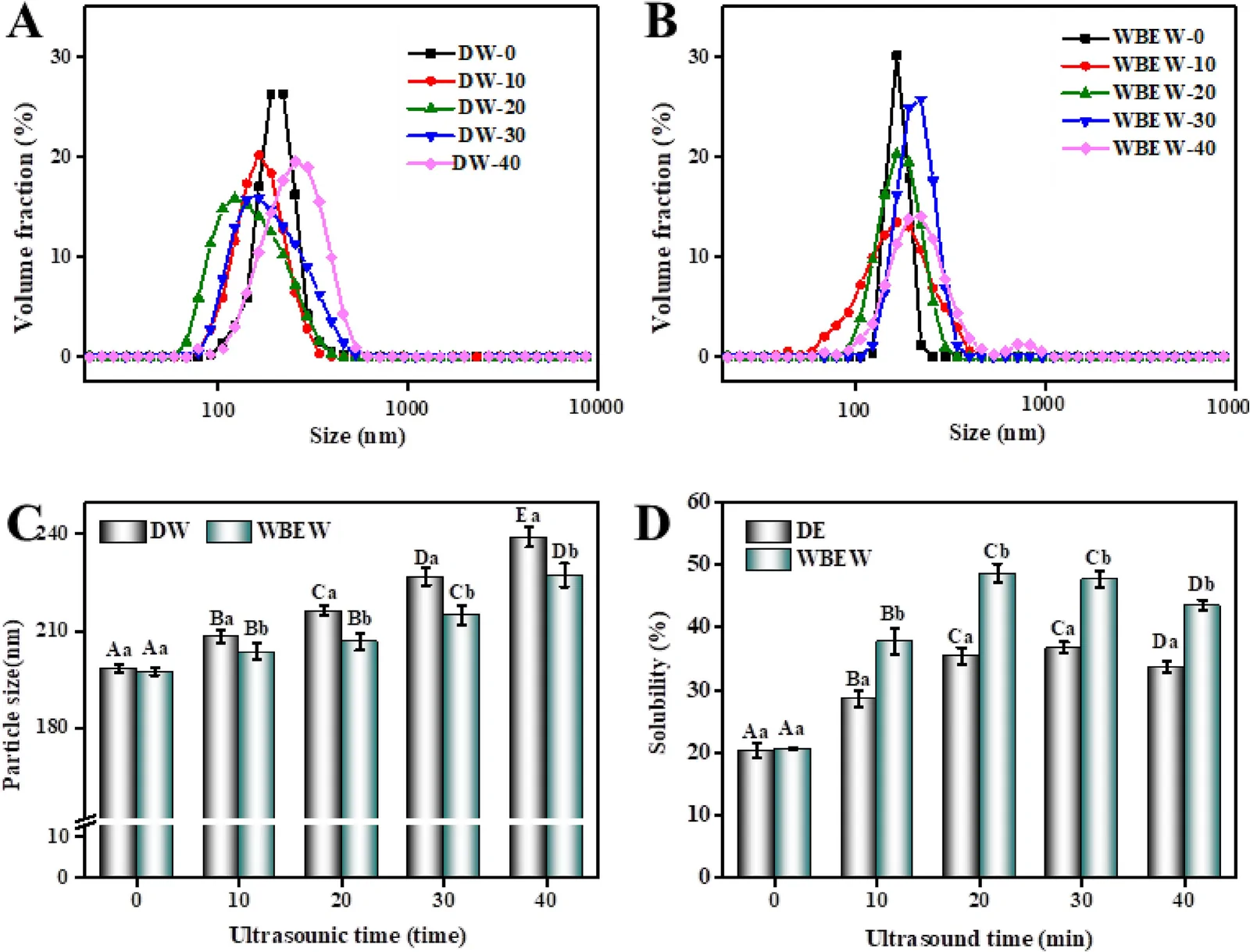
Effects of DW or WBEW coupled ultrasound treatment on the particle size (A*,* B and C) and solubility (D) of AKP.

Particle size and solubility are crucial physicochemical indicators that reflect protein aggregation state and hydration capacity. Fig. 2A–C illustrate the dynamic variations in AKP hydrodynamic particle size under different ultrasonic durations. In the DW group, the average particle size of native AKP was 216.4 nm at 0 min of sonication. As ultrasonic time extended to 20 min, particle size significantly decreased to 207.1 nm (*p* < 0.05), followed by a continuous upward trend when sonication exceeded 20 min. In comparison, the minimum particle size of WBEW-treated AKP reached 202.3 nm after 20 min of ultrasonic treatment, which was markedly smaller than that of the corresponding DW sample at identical time points. As reported by Jiang et al. [20], transient cavitation, microstreaming, and high-speed microjets generated by ultrasound can disrupt the intramolecular and intermolecular non-covalent interactions of proteins, including hydrophobic forces, electrostatic attraction, and hydrogen bonds. This action disassembles large native protein aggregates into smaller, dispersed fragments, thereby reducing the overall particle size. Nevertheless, excessively prolonged sonication reversed this trend, inducing a remarkable increase in particle size. This phenomenon arises from the massive sonogenerated ROS that trigger oxidative cross-linking between protein molecules via disulphide bonds, accelerating the reassembly of small protein fragments into large, insoluble aggregates [16]. Notably, AKP particles in all WBEW groups consistently maintained significantly lower average diameters than their DW counterparts at every ultrasonic time point, providing preliminary evidence that WBEW suppressed ultrasound-triggered protein oxidative aggregation by reducing radical accumulation.

Protein solubility results further corroborate the particle size trend. AKP solubility in both DW and WBEW systems exhibited a typical “rise-then-fall” profile with extended ultrasonic treatment (Fig. 2D). Moderate ultrasonic cavitation disrupted dense hydrogen-bonded protein aggregates, decomposed macromolecular assemblies into small, hydrated subunits, and improved the contact area between protein polar residues and water molecules, consequently elevating aqueous solubility. However, AKP solubility under WBEW treatment persistently surpassed that in DW samples across all sonication durations; the maximum solubility discrepancy of 26.5% was observed at 20 min of ultrasonic treatment for both media. Previous research suggests that excessive ROS accumulation during long-term sonication exacerbates protein side-chain oxidation and intermolecular covalent cross-linkage, forming hydrophobic macromolecular aggregates that weaken protein–water interaction and drastically reduce solubility [5], [21]. Owing to the robust reducing potential and radical-eliminating activity of WBEW, the generation of ·OH, singlet oxygen, and superoxide anions was substantially restrained during WBEW co-treatment, which retarded oxidative cross-linking and aggregate formation, thereby retaining superior hydration and AKP solubility.

**Fig. 3:**
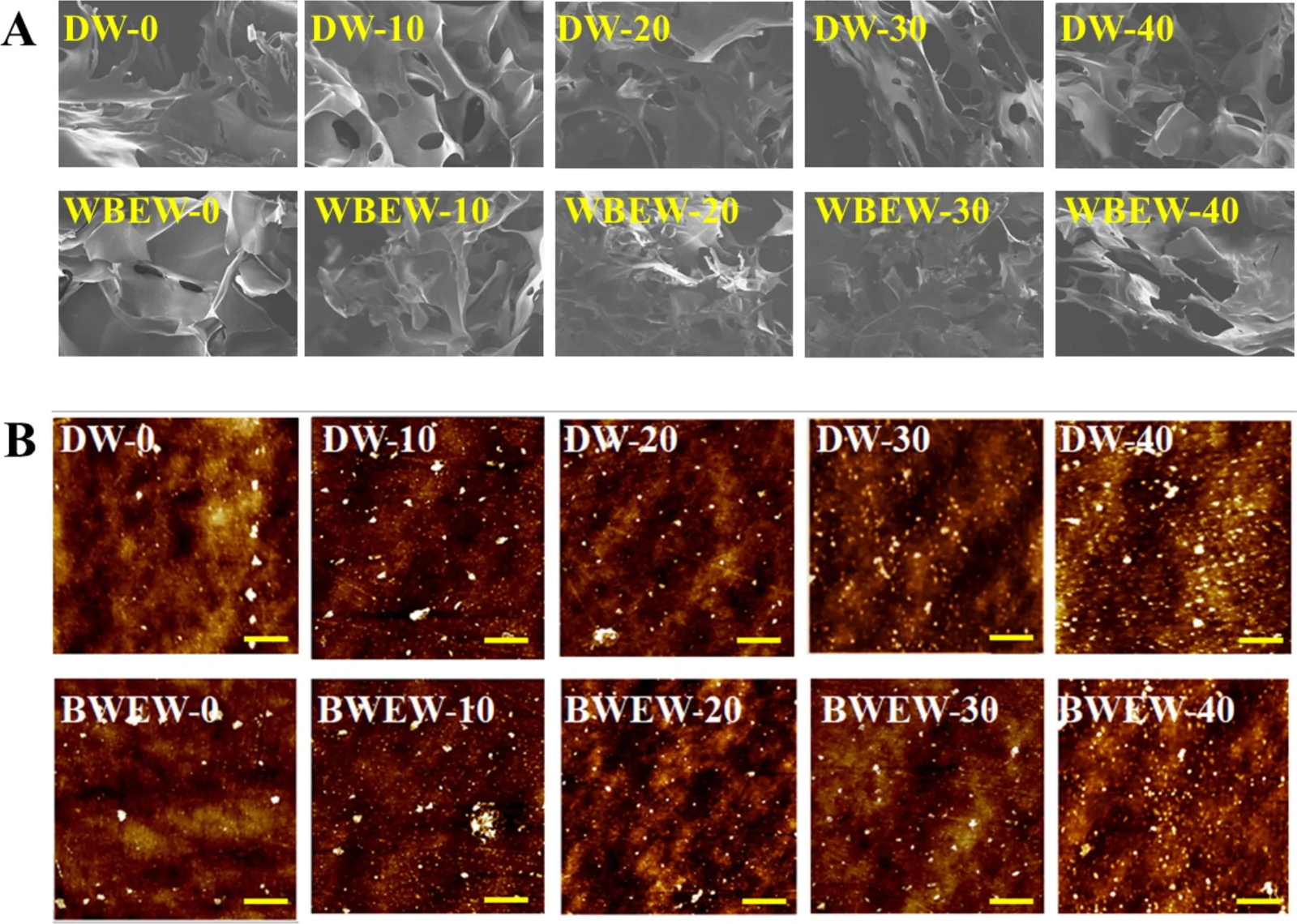
Effects of DW or WBEW coupled ultrasound treatment on the morphology (A: SEM, B: AFM) of AKP. Scale bar = 600 nm.

SEM was used to observe the micromorphology of freeze-dried AKP after DW and WBEW treatments (Fig. 3A). SEM micrographs revealed that untreated AKP tended to form irregular, fragmented, and disordered sheet structures. Short-duration sonication (< 20 min) in DW aggravated structural disorder, generating abundant thin, irregular flakes attributed to mechanical disaggregation of protein assemblies induced by ultrasonic shear force. In contrast, prolonged DW treatment (> 20 min) led to severe protein agglomeration, with massive compact, clumpy aggregates visible on the surface, which matched the increased particle size and decreased solubility data from DLS and solubility assays (Fig. 2). WBEW-treated AKP displayed analogous morphological variation trends to DW samples; however, the degree of large-scale aggregate formation was conspicuously alleviated at any matched ultrasonic time [11]. These intuitive microstructural observations directly verified that WBEW effectively mitigated ultrasonic oxidative damage to AKP, suppressed irreversible protein aggregation, maintained smaller particle size, and simultaneously improved aqueous solubility.

To overcome the inherent limitations of freeze-drying-induced artefacts in SEM imaging, AFM was employed to directly visualise the morphological features of AKP in a solution-deposited (Fig. 3B). The AFM images revealed that untreated AKP (DW-0) existed as relatively large, irregularly shaped particles with pronounced aggregation, consistent with the poor dispersibility of native AKP. After 20 min of ultrasonication in DW (DW-20), the particles became noticeably smaller and more uniformly dispersed, confirming that moderate cavitation forces effectively disrupted protein aggregates. However, prolonged ultrasonication (DW-40) induced severe re-aggregation, with the formation of large, clustered particles, providing direct evidence of oxidative cross-linking driven. WBEW-20 exhibited the smallest and most homogeneously dispersed particles, demonstrating that WBEW effectively mitigated ultrasound-induced oxidative damage. Even at 40 min, WBEW-40 maintained considerably finer dispersion than DW-40, confirming the sustained protective effect of WBEW's radical-scavenging capacity. These AFM observations are in excellent agreement with the DLS particle size measurements (Fig. 2A–C) and solubility data (Fig. 2D), providing evidence that WBEW effectively suppresses ultrasound-triggered protein oxidative aggregation and preserves a finely dispersed state in solution.

### 3.3 Structural properties of krill protein–GA complexes

**Fig. 4:**
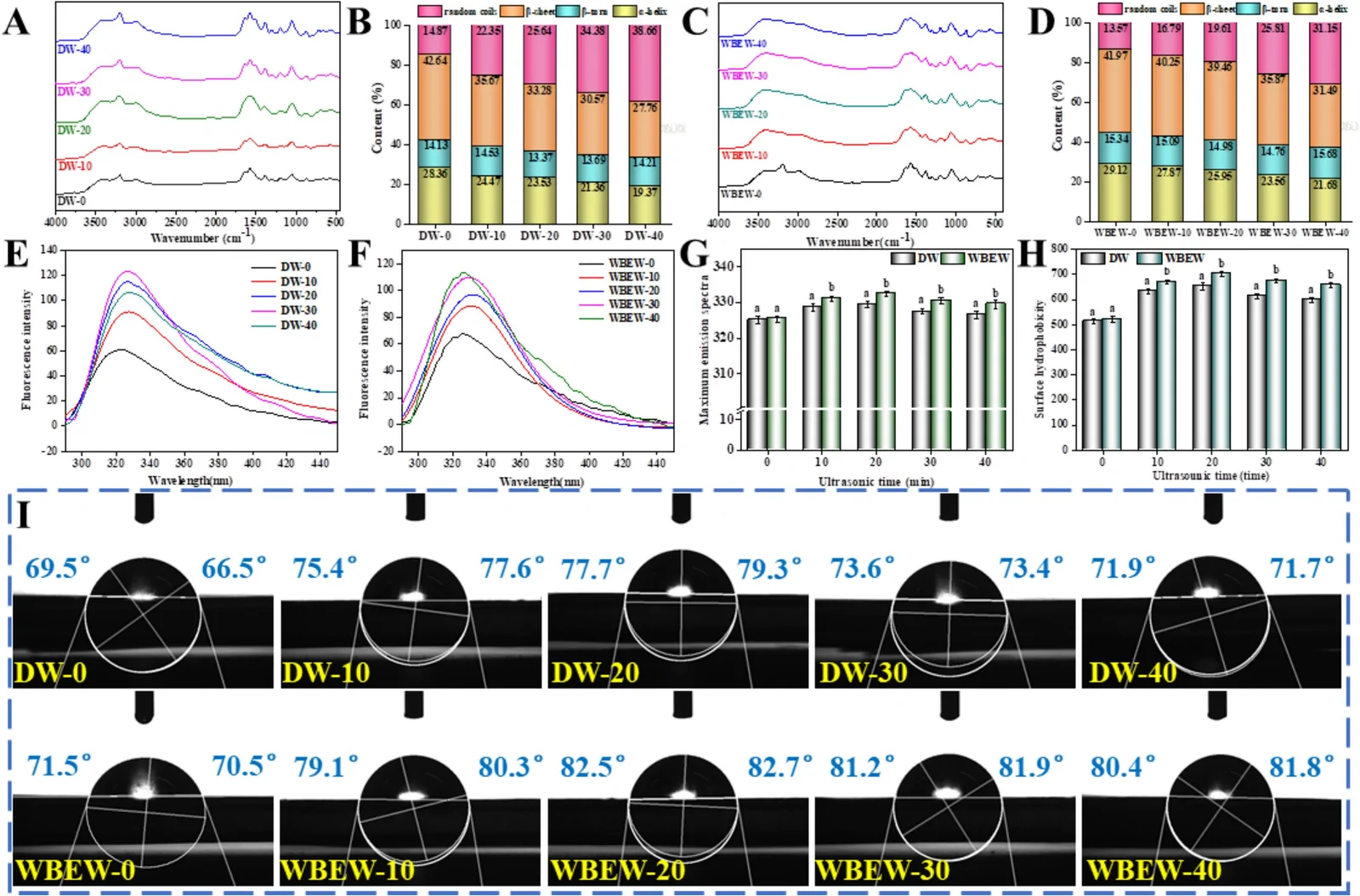
Effects of DW or WBEW coupled ultrasound treatment on the secondary structures (A −D), tertiary structure (E-G) and surface hydrophobicity (H-I) of AKPs.

FT-IR spectroscopy is a widely adopted technique for characterising the secondary structural rearrangement and molecular interactions of protein–polyphenol complexes [22]. The FT-IR spectra of AKP–GA complexes, treated with ultrasound in DW and WBEW, are presented in Fig. 4A and 4C. All samples exhibited characteristic protein absorption bands, corresponding to amides I and II, which are the most sensitive spectral regions for evaluating protein conformational changes [23]. Compared with that of the untreated control, the amide I peak of DW-treated AKP–GA complexes shifted from 1,639.18 to 1,646.27 cm^−1^. For the WBEW group, the amide I band shifted slightly from 1,640.24 to 1,644.57 cm^−1^. The displacement of amide characteristic peaks directly reflects alterations in protein skeleton bending and stretching vibration modes, as well as the rearrangement of intramolecular hydrogen bonds after modification [24]. Notably, the peak shift amplitude of the WBEW group was markedly lower than that of the DW group. This indicates that WBEW treatment effectively alleviated the drastic conformational perturbation of AKP–GA complexes induced by single ultrasonic treatment, maintaining a relatively stable protein skeleton structure [11], [18].

To quantitatively clarify the regulatory effect of different ultrasonic systems on protein secondary structure, the proportional modifications of α-helix, β-sheet, β-turn, and random coil conformations in AKP–GA complexes were further analysed. The untreated complex contained 28.36% α-helix, 42.64% β-sheet, 14.13% β-turn, and 14.87% random coil. After DW (Fig. 4B) and WBEW (Fig. 4D) treatment, both groups displayed consistent structural variation: the contents of ordered α-helix and β-sheet decreased significantly, while the proportion of disordered random coil increased remarkably. This phenomenon confirms that ultrasonic cavitation force disrupts the stable intramolecular hydrogen bonding network of proteins, transforming ordered compact secondary structures into disordered loose conformations [25].

Nevertheless, the reduction in α-helix and β-sheet content was considerably less pronounced in the WBEW group than in the DW group. Combined with the protein oxidation results, this structural difference can be plausibly explained: ultrasonically generated ROS induce protein carbonylation and amino acid oxidation, which destroy the stable spatial configuration of polypeptide chains and cause severe disorder of secondary structures [25]. The excellent free radical scavenging capacity of WBEW mitigates ultrasonic oxidative damage to AKP, thereby protecting the ordered secondary structure of protein–polyphenol complexes and minimizing structural disorder.

Tertiary structural changes in AKP–GA complexes were further investigated using endogenous fluorescence spectroscopy, a technique sensitive to the microenvironmental polarity of tyrosine (Tyr) and tryptophan (Trp) residues in proteins [26]. As depicted in Fig. 4E–G, both DW and WBEW treatments significantly augmented AKP’s endogenous fluorescence intensity compared with the untreated group. Moderate ultrasonic mechanical force unfolds the compact tertiary structure of proteins, thereby exposing buried endogenous fluorescent amino acid residues to the aqueous environment and increasing the fluorescence signal intensity. Fig. 4G illustrates the maximum emission wavelength (*λ_max_*) of the AKP–GA complexes. For the DW group, *λ_max_* red–shifted from 325.1  nm to 329.4  nm within 0–20  min of ultrasonication; when sonication time was further prolonged to 40  min, *λ_max_* blue–shifted to 323.6  nm. Similarly, in the WBEW group, *λ_max_* underwent an obvious red–shift from 325.5  nm to 334.4  nm during 0–20  min ultrasonication, followed by a blue–shift with further extended ultrasonic treatment. A red–shift in*λ_max_* signifies the unfolding of AKP’s tertiary conformation, leading to greater exposure of aromatic amino acid residues to the polar solvent. Conversely, prolonged ultrasonic treatment induces a blue–shift in *λ_max_*, which indicates oxidative re–aggregation of AKPs triggered by reactive radicals generated during extended sonication. This aggregation re–buries previously exposed fluorophores and results in the observed blue–shift [5]. It is worth noting that *λ_max_* values of the WBEW group were consistently higher than those of the DW group at identical ultrasonic durations, demonstrating a lower extent of oxidative protein aggregation in the WBEW system.

To further corroborate tertiary structural changes and variations in hydrophobic characteristics, *H_0_* and water contact angle were determined [12]. As illustrated in Fig. 4H, the *H_0_* values of all modified samples consistently increased initially, then decreased with ultrasonic duration, mirroring the *λ_max_* variation. Moderate ultrasonication unfolds the protein, exposing numerous hydrophobic amino acid residues on its surface and thereby enhancing surface hydrophobicity. However, excessive sonication exacerbates protein oxidative aggregation, leading to the re-covering of hydrophobic domains and a reduction in *H_0_*. Notably, WBEW groups maintained significantly higher *H_0_* than DW groups under identical treatment conditions, particularly during prolonged ultrasonication. Water contact angle tests further supported these findings (Fig. 4I). The contact angles for untreated DW and WBEW groups were 68° and 71°, respectively. After 20 min of ultrasonication, these values rose to 78.3° and 82.6°, subsequently decreasing with extended ultrasonic time. The higher contact angle of the WBEW group confirmed its superior surface hydrophobicity, indicating that WBEW effectively inhibited ultrasonic-induced oxidative re-aggregation of AKP and preserved more exposed hydrophobic active sites [5].

These results systematically elucidate the regulatory mechanism of WBEW treatment on AKP–GA complex structure. Moderate ultrasound unfolds the protein's ordered structures and exposes active binding sites. Concurrently, WBEW scavenges sonogenerated ROS, mitigating protein oxidative structural disorder and aggregation, thus maintaining a more stable and looser conformation of the complexes than single ultrasonic treatment. These favourable structural modifications are expected to underpin the improved functional properties of the complexes.

### 3.4 Functional properties of krill protein-GA complexes

**Fig. 5:**
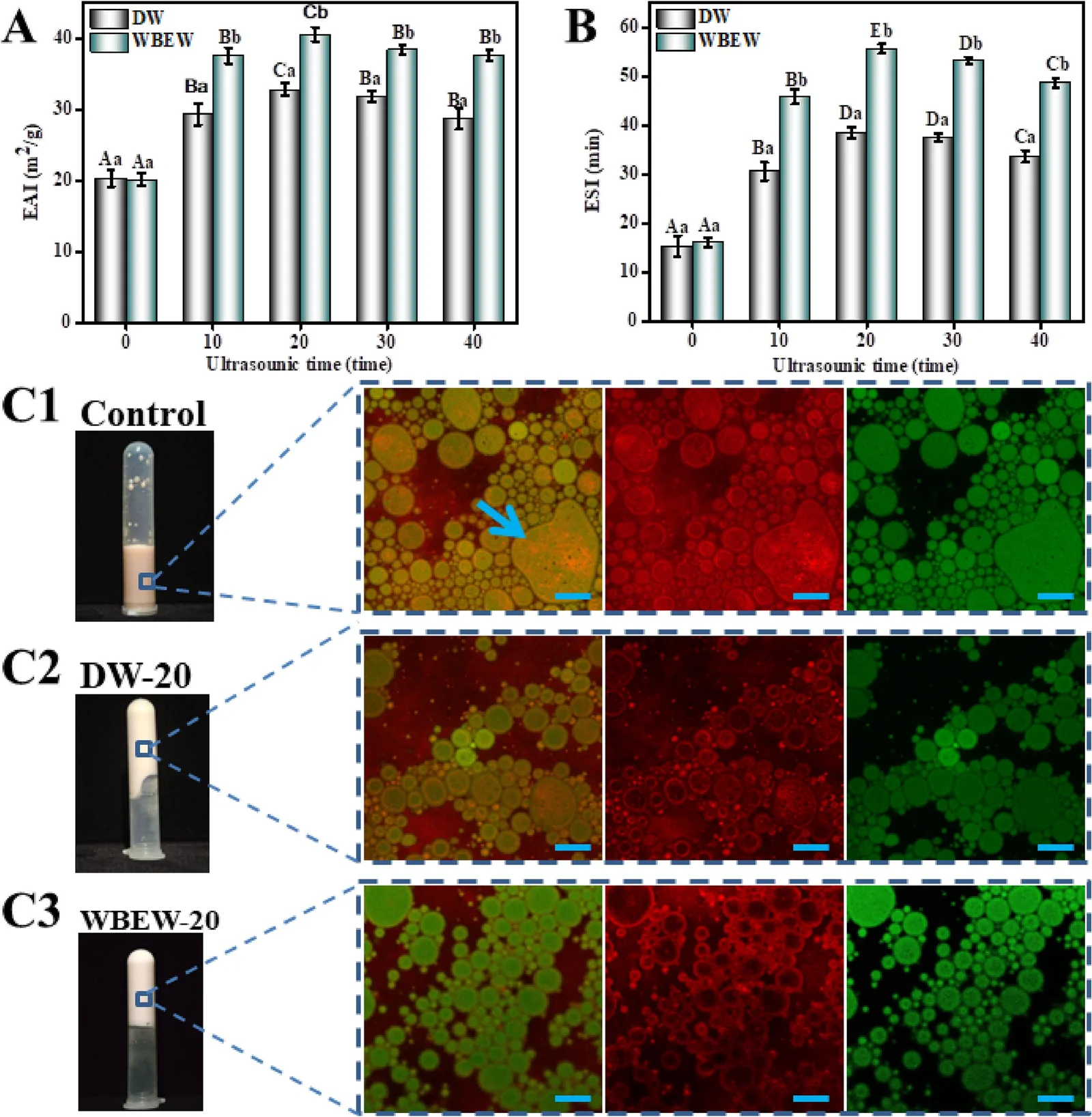
The emulsifying activity index (A) and emulsifying stability index (B) and CLSM images (C) of HIPEs stabilized by native AKP, AKP–GA complexes treated with ultrasound in DW or WBEW AKP–GA complexes. For CLSM images: green = protein (Nile Blue), red = fat (Nile Red). Scale bars = 530  μm.

Emulsifying activity index (EAI) and emulsifying stability index (ESI) are standard quantitative measures for assessing the interfacial adsorption capacity and phase separation resistance of protein emulsifiers, respectively [23], [27]. As illustrated in Fig. 5A, ultrasonic treatment significantly enhanced the EAI of AKP–GA complexes in both DW and WBEW systems (*p* < 0.05). The EAI initially increased and subsequently decreased with extended ultrasonic exposure. Moderate ultrasonic cavitation and mechanical shear forces unfold the compact tertiary structure of AKP, improving protein solubility and molecular flexibility, as well as exposing hydrophobic active sites. This leads to stronger interfacial adsorption and significantly enhanced emulsifying activity [28]. Notably, the WBEW group achieved a maximum EAI of 40.3 m^2^/g after 20 min of ultrasonic treatment, which was significantly higher than the 32.1 m^2^/g observed in all DW treatment groups under identical conditions. This optimal emulsification performance is linked to the structural protective effect of WBEW: WBEW treatment reduces excessive ultrasonically generated ROS levels, preventing severe oxidative aggregation of AKP and excessive degradation of GA. This maintains a moderately unfolded state of protein molecules, maximising the effective binding between AKP and GA. The resulting high-quality protein–polyphenol complexes exhibit optimised solubility and surface hydrophobicity, leading to excellent interfacial emulsification performance [29], [30]. Prolonged sonication, however, causes cumulative oxidative damage, forming protein cross-links and insoluble aggregates that diminish solubility and surface activity, thereby compromising emulsifying performance. Concurrently, excessive oxidation degrades the active phenolic hydroxyl groups of GA, attenuating the synergistic interfacial stabilisation effect of polyphenols and ultimately impairing the emulsifying activity of the complexes [31].

The ESI variation further confirmed WBEW’s regulatory effect on the emulsifying stability of AKP–GA complexes (Fig. 5B). The ESI of the DW group initially increased, peaking at 39.2 min after 20 min of ultrasonic treatment, then continuously decreased with prolonged sonication. Moderate ultrasonic modification promotes uniform adsorption of AKP–GA complexes on oil droplet surfaces, forming a continuous, dense interfacial protective film that effectively inhibits droplet collision and coalescence, thereby improving emulsion stability. Conversely, excessive ultrasonic oxidation induces protein aggregation and structural denaturation, resulting in loose, fragile interfacial films unable to resist droplet flocculation and phase separation, thus reducing emulsion stability. Importantly, the WBEW group maintained significantly higher ESI values than the DW group throughout the treatment period. This advantage stems from WBEW’s ability to inhibit ultrasonic oxidative damage, preserve the stable spatial structure of complexes, and ensure the structural integrity of interfacial adsorbed proteins. Furthermore, the high-efficiency binding of AKP and GA enhances the steric hindrance and intermolecular interaction of the interfacial film, further improving the physical stability of the emulsion system [30], [32].

The emulsifying property results, combined with the aforementioned findings, confirmed 20 min as the optimal ultrasonic treatment time for both systems. Consequently, the untreated control, DW-20, and WBEW −20 groups were selected for fabricating HIPEs. CLSM was subsequently used to visually confirm differences in emulsification performance. As depicted in Fig. 5C, the untreated control group (Fig. 5C1) failed to form a stable HIPE. CLSM images revealed extensive droplet aggregation, indicating inadequate interfacial stabilisation. While the DW-20-treated sample successfully formed a HIPE (Fig. 5C2), the emulsion displayed relatively poor stability, with noticeable coalescence and non-uniform droplet distribution. In contrast, the WBEW-20-treated sample produced a HIPE characterised by remarkably uniform droplet size, densely packed droplets, and a thick protein layer encasing the oil droplets (Fig. 5C3). The WBEW-20 complex's superior emulsion stabilisation capacity is primarily attributed to its favourable structural and physicochemical properties, including enhanced solubility, optimal surface hydrophobicity, improved interfacial activity, and resistance to oxidative aggregation. These properties facilitate the formation of a robust and cohesive interfacial film that effectively prevents droplet coalescence [33].

In summary, emulsifying property analyses indicate that WBEW-assisted ultrasound treatment effectively optimises the EAI and ESI of AKP–GA complexes, with 20 min identified as the optimal treatment duration. The WBEW-20 complex demonstrated superior emulsifying performance compared to its DW-20 counterpart, owing to enhanced solubility, favourable conformational features, and reduced oxidative damage. These findings suggest that the WBEW-20 complex possesses significant potential as a novel emulsifier for food applications.

### 3.5 Antioxidant properties of krill protein–GA complexes

**Fig. 6:**
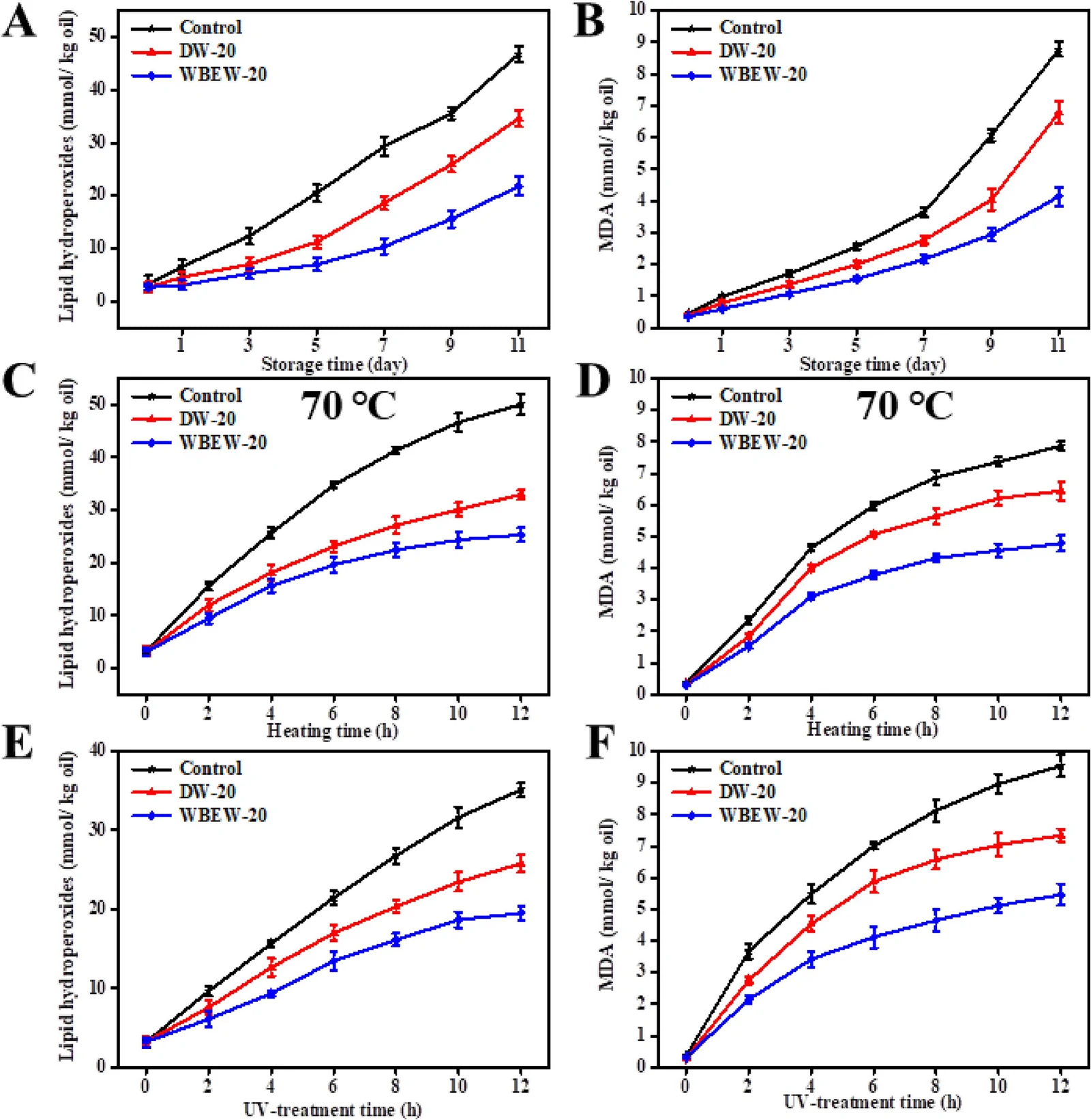
Concentration of primary reaction products (POV) and secondary reaction products (MDA) of HIPEs stabilized by native AKP, AKP–GA complexes treated with ultrasound in DW or WBEW AKP–GA complexes under long-term storage (A and B), heat (C and D) and UV light (E and F) treatment.

Peroxide value (POV) and malondialdehyde (MDA) are established, complementary indicators for assessing lipid oxidation. POV measures primary lipid hydroperoxides formed during initial oxidation, while MDA quantifies secondary decomposition products from later stages [11], [34]. Considering the optimal emulsification performance of DW-20 and WBEW-20-modified AKP–GA complexes, we systematically investigated the storage oxidation stability, thermal stability, and ultraviolet (UV) resistance of emulsions stabilised by various modified complexes. As displayed in Fig. 6A-B, all emulsion samples exhibited extremely low POV and MDA levels at the outset of storage (0 d), indicating no spontaneous lipid oxidation in freshly prepared emulsions. With prolonged storage, POV and MDA levels significantly increased in all groups (*p* < 0.05), demonstrating a consistent time-dependent oxidation trend. Notably, the degree of lipid oxidation in emulsions followed a distinct order: control group (emulsion stabilised by unmodified AKP) > DW-20 group (emulsion stabilised by AKP–GA complexes treated with ultrasound in DW) > WBEW-20 group (emulsion stabilised by AKP–GA complexes treated with ultrasound in WBEW). After 11 days of storage, the POV values for the control, DW-20, and WBEW-20 groups reached 43.6, 35.4, and 20.1 mmol/kg, respectively. Corresponding MDA values were 8.89, 6.97, and 4.34 mmol/kg. This significant difference indicates that single ultrasonic modification mildly improved the emulsion’s antioxidant capacity, whereas ultrasound coupled with WBEW treatment exhibited a superior synergistic antioxidant effect, effectively inhibiting lipid peroxidation in emulsion systems.

Furthermore, thermal treatment and UV irradiation are common external stressors that accelerate lipid oxidation during food processing and storage [35], [36]. In this study, emulsions subjected to thermal (Fig. 6C-D) and UV (Fig. 6E-F) treatment yielded comparable POV and MDA trends during long-term storage. The control group experienced the most severe oxidative deterioration under external stress, with rapid accumulation of primary and secondary lipid oxidation products. The DW-20 group provided limited antioxidant protection, while the WBEW-20 group consistently maintained the lowest POV and MDA levels under harsh thermal and UV conditions. These results confirm that WBEW-modified AKP–GA complexes impart excellent storage stability, alongside thermal tolerance and UV oxidation resistance to HIPEs, providing multi-scenario antioxidant protection for emulsion systems. The above findings demonstrate that the WBEW-20 complex effectively inhibited the formation of both primary and secondary lipid oxidation products in HIPEs during storage. We acknowledge that the present study did not experimentally isolate the individual contributions underlying this oxidative protection; accordingly, the following speculative but evidence-based hierarchy is proposed. (1) The antioxidant activity of GA bound to AKP is considered the primary contributor, as evidenced by the direct correlation between binding efficiency (Fig. 1F) and oxidative stability (Fig. 6), together with the well-established radical-scavenging and metal-chelating activities of protein-bound polyphenols at the oil–water interface [37]. (2) The WBEW-modified interfacial protein layer is regarded as a secondary but important contributor, since its denser, more continuous structure (3.2, 3.3, 3.4) physically impedes the diffusion of pro-oxidants into the oil phase, acting synergistically with bound GA [38]. (3) Residual reducing capacity derived from WBEW is deemed the least significant, as the extensive dialysis (48 h) and freeze-drying would largely eliminate the low-molecular-weight reducing species of freshly prepared WBEW, and any residual effect would be transient while the oxidative stability advantage persisted throughout the entire 11-day storage and under thermal/UV stress. Future work employing GA-free WBEW-treated AKP controls is warranted to quantitatively deconvolute these contributions. These findings underscore the potential of WBEW-assisted ultrasound treatment as a promising strategy for developing protein–polyphenol complexes with enhanced emulsifying and antioxidant functionalities for application in oxidation-sensitive food systems.

## 4 Conclusions

This study systematically investigated the effects of WBEW on ultrasound–assisted complexation between AKPs and GA, which extends our prior understanding of WBEW–ultrasound modification of pure krill protein. Different from previous work focusing on isolated protein, the current work focuses on protein–polyphenol composite system. WBEW effectively quenched ultrasound–generated ·OH radicals, reduced protein carbonyl formation and sulphydryl loss, and preserved GA integrity, thereby significantly enhancing AKP–GA binding efficiency. Optimal binding was achieved at 20 min of ultrasonication in WBEW, where the complex exhibited improved solubility, reduced particle size, and a more favourable conformational state with increased H*_0_*. These structural modifications translated into superior emulsifying properties, including higher EAI and ESI values, and enabled the formation of stable HIPEs with uniform droplet size and thick interfacial layers. Furthermore, the WBEW-20 complex effectively inhibited lipid oxidation during storage, as evidenced by significantly lower POV and MDA values. This was attributed to the synergistic effects of GA’s antioxidant activity, the interfacial barrier, and the residual reducing capacity of WBEW. Collectively, these findings demonstrate that WBEW assisted ultrasound treatment represents a promising modification strategy to improve functional performance and oxidative stability of AKP polyphenol complexes.

## CRediT authorship contribution statement

**Jinsong Wang:** Writing – review & editing, Resources, Investigation, Data curation. **Deyan Zhu:** Methodology, Investigation. **Rong Li:** Software, Formal analysis. **Yijiao Sun:** Investigation. **Yufeng Li:** Writing – review & editing, Validation, Methodology, Investigation.

## Declaration of competing interest

The authors declare that they have no known competing financial interests or personal relationships that could have appeared to influence the work reported in this paper.

## References

[1] Li Y., Zeng Q.-H., Liu G., Chen X., Zhu Y., Liu H., Zhao Y., Wang J.J. (2020). Food-grade emulsions stabilized by marine Antarctic krill (Euphausia superba) proteins with long-term physico-chemical stability. LWT-Food Sci. Technol..

[2] Li Y.F., Peng Z.Y., Tan L.J., Zhu Y.H., Zhao C., Zeng Q.H., Liu G., Wang J.J., Zhao Y. (2022). Structural and functional properties of soluble Antarctic krill proteins covalently modified by rutin. Food Chem..

[3] Wang Y., Xie Y., Wang A., Wang J., Wu X., Wu Y., Fu Y., Sun H. (2022). Insights into interactions between food polyphenols and proteins: an updated overview. J. Food Process. Preserv..

[4] Wang F., Li J., Qi Q., Mao Y., Yan X., Li X., Mu Y., Zhang H., Zhao C., Liu J. (2024). Structural, physicochemical and digestive properties of non-covalent and covalent complexes of ultrasound treated soybean protein isolate with soybean isoflavone. Food Res. Int..

[5] Pan J., Lian H., Jia H., Li S., Hao R., Wang Y., Zhang X., Dong X. (2020). Ultrasound treatment modified the functional mode of gallic acid on properties of fish myofibrillar protein. Food Chem..

[6] Wu P., Wang X., Lin W., Bai L. (2022). Acoustic characterization of cavitation intensity: a review. Ultrason. Sonochem..

[7] Pajares A., Bregliani M., Montana M.P., Criado S., Massad W., Gianotti J., Gutierrez I., Garcia N.A. (2010). Visible-light promoted photoprocesses on aqueous gallic acid in the presence of riboflavin. Kinetics and mechanism. Journal of Photochemistry and Photobiology a-Chemistry.

[8] Li Y., Wang J., Zeng Q.-H., Wang L., Wang J.J., Li S., Zhu J., Zeng X.-A. (2024). Novel thawing method of ultrasound-assisted slightly basic electrolyzed water improves the processing quality of frozen shrimp compared with traditional thawing approaches. Ultrason. Sonochem..

[9] Rahman S.M.E., Khan I., Oh D.-H. (2016). Electrolyzed water as a novel sanitizer in the food industry: current trends and future perspectives. Compr. Rev. Food Sci. Food Saf..

[10] Lee M.Y., Kim Y.K., Ryoo K.K., Lee Y.B., Park E.J. (2006). Electrolyzed-reduced water protects against oxidative damage to DNA, RNA, and protein. Appl. Biochem. Biotechnol..

[11] Li Y., Wang J., Zhu X., Tan L., Xie D., Xu W., Gui Y., Zhao Y., Wang J.J. (2022). Basic electrolyzed water coupled with ultrasonic treatment improves the functional properties and digestibility of Antarctic krill proteins. Food Res. Int..

[12] Lu H., Yang S., Chen J., Zeng S., Chen H., Zheng B. (2026). Ultrasound-assisted modification of lotus seed protein: Structural and functional enhancements. Int. J. Biol. Macromol..

[13] Li B., Zhong X., Zhang X.L., Zhang Y.X., Gao X.H., Liu X.M., Su W.T., Wang Y.X. (2025). Ultrasonic-assisted Haematococcus pluvialis protein-pectin complex significantly enhances DHA stability and bioavailability for 3D food printing. Food Chem.-X.

[14] Shi L.S., Yang X.Y., Gong T., Hu C.Y., Shen Y.H., Meng Y.H. (2023). Ultrasonic treatment improves physical and oxidative stabilities of walnut protein isolate-based emulsion by changing protein structure. LWT-Food Sci. Technol..

[15] Yang H., Xu F., Wang H., Ji L., Shen Q. (2025). Structural and morphological optimization of low-salt myofibrillar proteins aggregates by ultrasound treatment: modulation of thermal aggregation behavior. Food Chem..

[16] Kang D.-C., Zou Y.-H., Cheng Y.-P., Xing L.-J., Zhou G.-H., Zhang W.-G. (2016). Effects of power ultrasound on oxidation and structure of beef proteins during curing processing. Ultrason. Sonochem..

[17] Deng Q., Chen Z., Lao S., Min D., Liu X., Jiang H. (2025). Impacts of ultrasound-assisted alkaline electrolyzed water extraction on efficiency and bioactivity of extracts from sweet tea. Food Anal. Methods.

[18] Iscimen E.M., Capar T.D., McClements D.J., Yalcin H., Hayta M. (2023). Ultrasound-assisted preparation of faba bean protein isolate-*Vitis vinifera* L. polyphenol extract conjugates: Structural and functional characterization. Food Biosci..

[19] Zhang Q., Li H., Cen C., Zhang J., Wang S., Wang Y., Fu L. (2021). Ultrasonic pre-treatment modifies the pH-dependent molecular interactions between β-lactoglobulin and dietary phenolics: Conformational structures and interfacial properties. Ultrason. Sonochem..

[20] Jiang Y.-H., Huang P.-M., Li Y.-Y., Zhao Y.-T., Zheng Y., Zhou Y.-X., Xin W.-G., Suo H.-Y. (2026). Insights into the effects of ultrasound on the structural, aggregation behavior, and functional properties of almond isolate protein: an integrated experimental and molecular dynamics simulation studies. Food Res. Int..

[21] Chen J., Chen X., Zhou G., Xu X. (2022). New insights into the ultrasound impact on covalent reactions of myofibrillar protein. Ultrason. Sonochem..

[22] Kadam A., Elhordoy M.R., Vazquez D., Medrano A., Gough K.M., Koksel F. (2026). Molecular insights into soy protein-lupin flour meat analogues through FTIR spectrochemical analysis. J. Agric. Food Chem..

[23] Li Y., Wang J., Wu Y., Guo S., Zhu L., Ou X., Li T., Zhao Y., Wang J.J. (2025). Effects of ultrasound combined with pH-shift assisted glycosylation modification on the structural and functional properties of by-product proteins extracted from shrimp heads. Food Chem.-X.

[24] Wang X., Ni X., Duan C., Li R., Jiang X., Xu M., Yu R. (2024). The effect of ultrasound treatment on the structural and functional properties of tenebrio molitor myofibrillar protein. Foods.

[25] Baltacioglu H., Bayindirli A., Severcan F. (2017). Secondary structure and conformational change of mushroom polyphenol oxidase during thermosonication treatment by using FTIR spectroscopy. Food Chem..

[26] Alondra Cuevas-Acuna D., Luis Arias-Moscoso J., Torres-Arreola W., Cadena-Cadena F., Gertrudis Valdez-Melchor R., Chaparro-Hernandez S., del Carmen Santacruz-Ortega H., Ruiz-Cruz S. (2020). High-intensity ultrasound pulses effect on physicochemical and antioxidant properties of tilapia (Oreochromis niloticus) skin gelatin. Appl. Sci.-Basel.

[27] Fan Y., Peng G., Pang X., Wen Z., Yi J. (2021). Physicochemical, emulsifying, and interfacial properties of different whey protein aggregates obtained by thermal treatment. LWT-Food Sci. Technol..

[28] Ding Q., Ranasinghe R.A.D.A., Mo Z., Yang H., Luo L., Ma H., Li X., Dai C., Zhang T. (2026). Ultrasound-induced modifications of fish proteins: a comprehensive review of structural, physicochemical, functional properties, and industrial applications. Food Res. Int..

[29] Xu B., Dang L., Zhang Y., Wei Z., Wang L., Ma B., Zheng Y., Li J., Shang S. (2026). Mechanism underlying the enhancement of emulsifying properties of millet bran prolamine via ultrasound-assisted gallic acid covalent-binding or carboxymethylation under pH-shifting. Int. J. Biol. Macromol..

[30] Tran Hong Q., Benjakul S., Sae-leaw T., Balange A.K., Maqsood S. (2019). Protein-polyphenol conjugates: antioxidant property, functionalities and their applications. Trends Food Sci. Technol..

[31] Huang Y., Zheng B., Sun B., Zhu Y., Liu L., Liu J., Zhang Y., Li Y., Zhu X. (2025). Mechanism of emulsion stabilized by an ultrasonically prepared protein-polyphenol-polysaccharide complex: structure, functional properties and interfacial behavior. Ultrason. Sonochem..

[32] Gong X.X., Sun Q.C., Wang X.Y., Zhang R.H., Peng Y., Cui L.Q. (2025). Recent advances in pulse protein conjugation and complexation with polyphenols: an emerging approach to improve protein functionality and health benefits. Crit. Rev. Food Sci. Nutr..

[33] Mourad F.K., Cai Z. (2025). Ultrasound-assisted egg yolk granule-EGCG conjugation via multiple mechanisms for improved functionality and stability. Food Chem..

[34] Zhang M., Fan L., Liu Y., Li J. (2023). Migration of gallic acid from the aqueous phase to the oil-water interface using pea protein to improve the physicochemical stability of water-in-oil emulsions. Food Hydrocoll..

[35] Chenah M., Karabulut G., Zannou O., Tosif M.M., Goksen G. (2026). Multi-component chitosan-pectin films reinforced with Padina pavonica nanocellulose and Thymus algeriensis essential oil for red meat shelf-life enhancement. Food Chem.-X.

[36] Moll E., Chiralt A. (2025). Active PHBV films with ferulic acid or rice straw extracts for food preservation. LWT-Food Sci. Technol..

[37] Guo X., Wei Y., Liu P., Deng X., Zhu X., Wang Z., Zhang J. (2024). Study of four polyphenol-Coregonus peled (C. peled) myofibrillar protein interactions on protein structure and gel properties. Food Chem.-X.

[38] Nie X., Zhao L., Wang N., Meng X. (2017). Phenolics-protein interaction involved in silver carp myofibrilliar protein films with hydrolysable and condensed tannins. LWT-Food Sci. Technol..

